# Distributed Adaptive Search in T Cells: Lessons From Ants

**DOI:** 10.3389/fimmu.2019.01357

**Published:** 2019-06-13

**Authors:** Melanie E. Moses, Judy L. Cannon, Deborah M. Gordon, Stephanie Forrest

**Affiliations:** ^1^Moses Biological Computation Laboratory, Department of Computer Science, University of New Mexico, Albuquerque, NM, United States; ^2^Biology Department, University of New Mexico, Albuquerque, NM, United States; ^3^Santa Fe Institute, Santa Fe, NM, United States; ^4^The Cannon Laboratory, Department of Molecular Genetics & Microbiology, University of New Mexico School of Medicine, Albuquerque, NM, United States; ^5^Department of Pathology, University of New Mexico School of Medicine, Albuquerque, NM, United States; ^6^Autophagy, Inflammation, and Metabolism Center of Biomedical Research Excellence, University of New Mexico School of Medicine, Albuquerque, NM, United States; ^7^Department of Biology, Stanford University, Stanford, CA, United States; ^8^Biodesign Institute and School for Computing, Informatics, and Decision Sciences Engineering, Arizona State University, Tempe, AZ, United States

**Keywords:** T cells, ant foraging, adaptive search, collective search, ant inspired algorithms

## Abstract

There are striking similarities between the strategies ant colonies use to forage for food and immune systems use to search for pathogens. Searchers (ants and cells) use the appropriate combination of random and directed motion, direct and indirect agent-agent interactions, and traversal of physical structures to solve search problems in a variety of environments. An effective immune response requires immune cells to search efficiently and effectively for diverse types of pathogens in different tissues and organs, just as different species of ants have evolved diverse search strategies to forage effectively for a variety of resources in a variety of habitats. Successful T cell search is required to initiate the adaptive immune response in lymph nodes and to eradicate pathogens at sites of infection in peripheral tissue. Ant search strategies suggest novel predictions about T cell search. In both systems, the distribution of targets in time and space determines the most effective search strategy. We hypothesize that the ability of searchers to sense and adapt to dynamic targets and environmental conditions enhances search effectiveness through adjustments to movement and communication patterns. We also suggest that random motion is a more important component of search strategies than is generally recognized. The behavior we observe in ants reveals general design principles and constraints that govern distributed adaptive search in a wide variety of complex systems, particularly the immune system.

## Introduction

T cells are key players in adaptive immunity, required for clearance of virally infected cells and tumor cells. Improved understanding of how T cells search may lead to more effective T cell vaccine design and cancer immunotherapies. Many types of immune cells search for pathogens or other targets, but T cell search is especially challenging because T cells are often responding to novel pathogens. Ant colonies are another distributed adaptive system in which individual agents search cooperatively, without centralized control, to find targets in unknown locations in a complex environment. However, ant colonies are simpler and, in some ways, easier to observe than immune systems. Here we propose new hypotheses about how T cells search suggested by successful search strategies in ant colonies.

T cells search for many kinds of targets, at many scales, and in many different tissues including lymph nodes, infected tissues and systemic infection in the whole body. Prior to infection, T cells migrate through the lymphatic network to search within lymph nodes for potential activating antigen presented by dendritic cells (1, 2). If a naive T cell finds a dendritic cell bearing its cognate antigen in the lymph node, the T cell then proliferates and migrates out of the lymph node through the cardiovascular network, extravasating at the site of infection in peripheral tissue, where activated T cells conduct a second search to find and eliminate target cells (3).

How do the interactions among T cells, host cells, target cells, and tissue architecture generate the remarkably rapid and effective immune response to a wide variety of pathogens and tumors? Computational and mathematical approaches have described aspects of immune responses (4, 5) including the T cell repertoire (6), development of the effector and memory responses (7–11), and T cell responses to infections including HIV (12), influenza (13), and anti-tumor responses (14–16) just to name a few. Mathematical models have been developed to study how T cell movement through lymph nodes impacts T cell activation (17–22). However, relatively few mathematical models have connected individual T cell movement and interactions during search to the broader outcomes of immune response to infection, particularly in complex tissue environments (19, 23, 24).

Foraging strategies in ants suggest a framework for understanding how collective search strategies emerge from the behaviors of individual agents (25–27). These questions are difficult to answer experimentally in immunology, especially at the scale of an entire organ or body. In ant colonies, we can simultaneously observe the small-scale behavior of individuals and the large-scale collective, such as shifts in the allocation of ants to various tasks (28), territorial interactions between different colonies, (29–31), and the recruitment of searchers to discovered food (32–34). Thus, extrapolating understanding about the search strategies of ants to immune responses can suggest general design principles that can then be tested in the immune system.

We use the understanding of ant foraging gained from experiments and models to provide insight into T cell search processes. We find that there are significant parallels between how ants forage for food and how T cells search for pathogens. First, both T cells and ants combine random movement with directed movement to produce an effective search strategy across a wide variety of environmental conditions. Both ants and T cells search for targets whose positions are unknown, dispersed, and can be both mobile and ephemeral, thus ants and T cells need random elements in their strategies to flexibly adapt to dynamic conditions and varied environments. Second, ants and T cells both use communication to improve search efficiency by following chemical signals to the locations of their targets; additionally, direct agent-agent interaction may provide a direct form of communication to increase search efficiency. Third, physical structures, such as nest and trail structure for ants and the lymphatic network and the stromal cell network in tissues, provide spatial networks embedded in the search space that can guide the movement of searchers.

Studies of ant foraging reveal that effective search strategies incorporate an appropriate balance of movement that is random, guided by signals and agent interaction, and mediated by traversal of physical structures. We focus on how the appropriate balance depends on two factors. First, the best search strategy depends on the distribution of targets in time and space. Second, the best strategy depends on whether the objective of the search is to be fast (finding targets as quickly as possible) or complete (finding all available targets), or some combination of the two. Search strategies from ant foraging suggest specific hypotheses that can be tested to reveal novel search strategies taken by T cells in complex tissue environments leading to more efficient immune responses.

## Ant Foraging as a Model for T Cell Search

Ant colonies are a canonical example of collective intelligence, demonstrating strategies for effective distributed search in varied ecological spaces in almost every terrestrial habitat on Earth. Each of the 14,000 species of ants has evolved in a particular environment, leading to diversity among species in how they move, interact with each other and use physical structures as they forage for food. Ecological and evolutionary studies show a correspondence between foraging behavior and the dynamics of the resources that a species uses (34–37). The Lanan review thoroughly catalogs a remarkable diversity of foraging strategies, including different forms of movement, recruitment and trail formation, and shows that different environmental conditions faced by different species generate predictable regularities in these foraging strategies.

**Figure 1:**
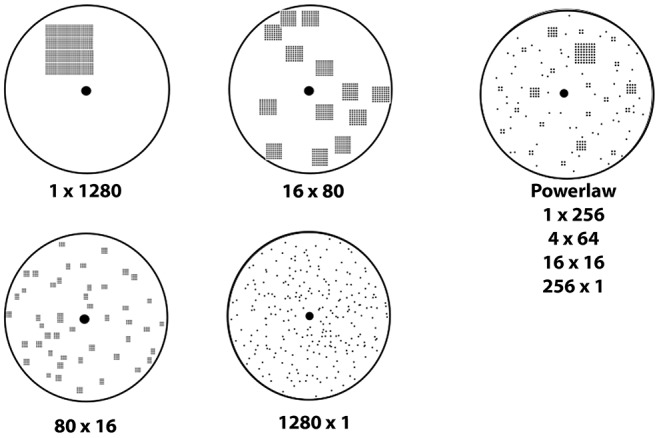
Target distributions showing a range of clusteredness from a single pile of 1280 targets to 1280 targets distributed at uniform random. Each shows the number of piles and the number of targets per pile. The power law distribution has a mix of pile sizes with the number of piles inversely related to pile size.

**Figure 2:**
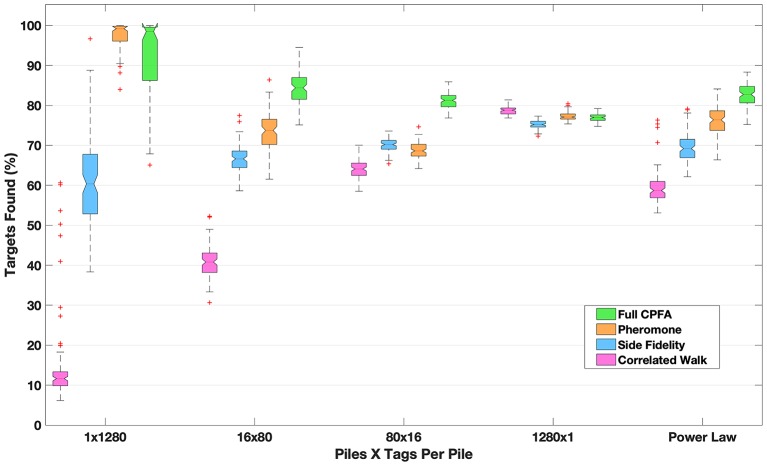
The number of targets found in a 1 h simulation given different search strategies and target distributions (shown in Figure 1). For the most clustered distribution, pheromone recruitment to piles vastly outperforms random search (a CRW). The relative performance of pheromone recruitment compared to random search declines as targets are more dispersed. Site fidelity performs better than random search but not as well as pheromones in all of the clustered distributions. All strategies perform approximately equally for targets dispersed uniformly at random (1280 x 1). In the full CPFA, searchers choose whether to use random search, site fidelity or pheromone recruitment depending on the size of the piles they sense while searching. It is the most effective search strategy across all distributions, and it most clearly outperforms other strategies given intermediate pile numbers and sizes (e.g., 80 × 16) and the power law distribution which has mixed pile sizes.

A key feature that influences search strategy is the distribution of resources in time and space (36, 38–40). Targets can be patchy, clustered into one location, or dispersed uniformly at random through the entire search area. Figure 1 shows examples of spatial distributions from highly clustered to highly dispersed, as well as a power law distribution with both clusters and dispersed targets. Models of ant foraging have demonstrated that the speed of target collection changes dramatically depending on how targets are distributed [Figure 2, discussed in section IV, (39, 41)]. For example, when resources are patchy, foragers recruit each other to the location where resources have been found. One mechanism for recruitment is chemical pheromone trails that, by inducing one ant to follow another, generate information about the location of food (34, 40, 41). By contrast, when resources are scattered or ephemeral, pheromone recruitment is pointless, and ants do not guide each other in any particular direction [instead regulating whether or not to forage at all, (42)].

The objective of the search also plays a role in determining the most effective search strategy. We highlight two such objectives. *Fast detection* weights detection speed of the first targets most heavily, while for *complete detection* the goal is to find all of the targets. Some searches combine these objectives, i.e., finding all targets as quickly as possible, but in many instances either speed or completeness is deemphasized. Our computational models show that the nature of the search problems matters: successful strategies for complete detection differ from those for fast detection (43–45).

Ant colonies provide several examples of fast detection. In foraging by desert seed harvester ants (one of most well-studied ant groups), the goal is to collect as many resources as possible in a fixed time window. The foraging window is limited because ants lose water rapidly while foraging in the hot sun (46), so the search must be fast. However, seeds remain on the ground and in the soil for a long time, so it is not important to collect all available seeds immediately, as they will be available later. Our models, described below, show that ants that recruit each other to a single pile may also achieve complete collection of that pile, but when resources are dispersed among many piles, the ant strategies we model fail to achieve complete collection, for example taking much longer to find the last 10% of targets than the first 90% (43).

The stability of targets over time also influences both fast and complete detection. For example, recruitment of agents to a particular location is useful only if targets persist long enough in one place for other searchers to find the targets when they arrive (34, 40). Consequently, ants that forage for resources that are both clustered in space and persistent in time evolve strategies for recruitment, and ants that forage for randomly dispersed resources do not.

## T Cell Search Must be Fast, and Often Must be Nearly Complete

Our characterization of ant search suggests new ways to interpret search behaviors of T cells. T cells in the lymph node search for antigen presented by dendritic cells. To succeed in initiating the adaptive immune response quickly, this search must be fast rather than complete. Speed is important because pathogen replication is an exponential process. However, because multiple antigens can be presented to multiple T cells, T cells do not need to interact with every possible DC. Instead, T cell search in the lymph node needs to detect only enough antigen to initiate activation. This search problem resembles our models of ants conducting fast, but not complete, searches for dispersed seeds.

In contrast, for T cell search in the periphery, thoroughness, or complete detection, is crucial in some cases. T cells must detect and eliminate virtually all pathogens. In influenza, for example, successful control requires finding and eliminating all, or nearly all, influenza virus in the lung. Similarly, in immunotherapy, the goal is for effector CD8 T cells to identify and kill all viable tumor cells. Our ant models quantify how this complete detection task becomes easier when targets are clustered in one or a few places and becomes more challenging when they are dispersed broadly.

Some immunological studies have described target distributions (10, 47, 48); however, models of the immune response rarely consider how effective different search strategies are at finding targets with different spatial distributions. Very little is known about how long different targets such as pathogens or tumor cells might persist in specific tissue locations or how mobile pathogens might be within tissues. Our ant foraging models suggest that understanding target distribution in tissues may be an important determinant of effective T cell search and immune responses.

## Ant-inspired Hypotheses About T Cell Search

### Random and Directed Motion Combine to Produce Effective Search

Ants foraging for food often use a form of random search called a correlated random walk (CRW) (49–51). In a CRW, the angle of each successive turn is correlated with that of the previous one, and movement patterns are straighter and less convoluted than in Brownian motion. CRWs are more dispersive than Brownian motion, thus increasing the physical extent of the search area while decreasing its thoroughness by minimizing repeated sampling of the same area. In models designed to maximize the speed at which seeds are detected by foraging ants, a high degree of correlation among steps (leading to more straight-line ballistic motion) appears optimal for fast detection (39, 52, 53).

When the location of targets is known with sufficient probability, ants can move directly toward the location de-emphasizing random movements. For example, ants use a process called site fidelity to return repeatedly to the location of a previously found a seed (54, 55). Beverly et al. found that when an ant finds a seed, it returns directly to that location with over 90% probability. Other species use site fidelity to search for resources that are clustered, even if those clusters are small or variable in size. Recruitment through olfactory interactions based on pheromones is a well-known mechanism by which ants attract other ants to locations where food is abundant or persistent.

The overwhelming complexity of the immune system reflects the different kinds of problems it is required to solve. For example, in some cases immune cells must search broadly for rare targets, and in other cases it must search thoroughly to find all targets. We used models of random and directed motion developed for ants to analyze how T cells move, building on existing studies of T cell motion in tissues. Initial work suggested that naive T cells in lymph nodes move randomly, and models of T cell movement assumed Brownian motion of T cells to estimate how many T cells are required to find DCs (8, 56). More recently, researchers hypothesized that T cell movement is characterized by Levy walks, another form of random motion in which cells move in random direction for multiple time steps drawn from a power law distribution (57). We demonstrated that prior to infection, both CD4 and CD8 T cell movement in the lymph node is random, but does not follow idealized Brownian or Levy movement patterns (58). Instead, our models show that T cells can disperse more quickly compared to Brownian motion, leading to more effective search for DC targets in lymph nodes. Our model predicts that the particular movement pattern we observe in T cells (a CRW with step lengths drawn from a lognormal distribution) balances thorough search in a small region with extensive search in a broader area. We hypothesize that these ant-like movement types affect how quickly T cells encounter rare vs. abundant antigen in the lymph node.

T cell motion in infected tissues varies according to the requirements of the task being performed. It is clear that migration of effector T cells into infected tissue is signal dependent and directional toward areas of inflammation, including in skin (24), brain (57), lung (59), vaginal tract, and gut (3). However, some studies suggest that once T-cells reach skin tissue their movement within the tissue is not highly directional toward the foci of infection (24). Our work found that effector T cells in inflamed lung also move in a CRW, similar to naive T cells in lymph node, suggesting random motion. In the lung, the T cell CRW is combined with a stop-and-go mode of intermittent motion, which enables effector T cells to search a larger area while also interacting with potential target cells (60). In contrast to cells in the lung, Harris et al. found that effector T cells in the brain of Toxoplasma infected animals move with a Generalized Levy walk (57). And, there is no evidence in brain or lung tissue that effector T cells move directionally toward sites of infection. Ant search models suggest one hypothesis to explain the lack of directional movement: if sites of infection are usually dispersed in space, for example, in tissues, T cells may have evolved search strategies to explore broadly for new sites of infection rather than focusing on exploiting already detected foci of infection.

### Physical Structures for Effective Search

Networks provide physical structures that can increase search efficiency, minimizing the distance traveled to explore large spaces. Ants use environmental structures to extend their search. For example, turtle ants create trail networks within the network of vines and branches in the canopy of the tropical forest (26, 27). In some ant species, ants search along edges such as cracks in sidewalks and search from these main trails (61), apparently using environmental structures to explore the environment but not necessarily moving directly toward food sources. We suggest that one role of structural networks is to enhance the scalability of search to larger physical spaces.

Recent work imaging T cells in intact tissues suggests that T cells may also use structural networks to mediate motion. At the organism scale, cardiovascular and lymphatic networks disperse immune cells throughout all tissues to enhance response to infection anywhere in the animal. Effector cells in skin were shown to move along collagen fibers (62), and effector T cells in inflamed lung move along vasculature (60). Within the lymph node, T cells use fibroblastic reticular cells (FRC) as guidance cues (63, 64). The FRC network in the lymph node has the topological structure of a small world network, which likely enhances the robustness of T cell responses to damage to the network (65, 66). Small world networks with many local and a few long-distance connections significantly increase scalability, cohesion, and efficiency of exploration via the network (67).

T cell movement along tissue structures resembles that of ants traveling along branches. T cell movement along structures such as collagen, vasculature, and FRCs does not obviously lead to targets (64). There is also no evidence that effector T cells in lung and skin, where directional motion is important, use structural guidance to travel toward targets. Instead of providing directional guidance toward targets, we suggest that movement along networks may instead enhance scalability and maximize exploration of large spaces.

### Distributed Communication: Soluble Signals and Direct Agent Contact

A striking similarity between ant colonies and immune systems is the use of chemical signals for communication. It is well-established that both systems use chemical cues to signal the presence of danger: alarm pheromone in the case of ants, cytokines in the case of immune systems. Both systems also use chemical signals to recruit other agents to search more effectively: immune cells can follow chemokine gradients to sites of infection, much like ants can follow pheromone trails to food.

Ants use pheromones to create dynamic maps. They do so by laying pheromone trails from locations with abundant food back to the nest, a form of communication through the environment, known as stigmergy (68). Such pheromone trails encourage other ants to travel directly to the food source, reinforcing the trail if they find food successfully. Once the food is depleted, the ants stop reinforcing the pheromone trail, and over time it dissipates and ceases to attract new ants to that location. This process is well-studied both experimentally in laboratory and field studies of various ant species, and in mathematical and computational models [as reviewed in (69)]. It is also the basis of a popular computational problem-solving heuristic called Ant Colony Optimization (70). These studies reveal the benefits and limits of pheromone communication in search problems, providing a roadmap for immunologists to understand how chemokines influence search.

A variety of chemical signals guide movement of immune cells, particularly to sites of infection. For example, chemokines provide migration and localization signals to dendritic cells, neutrophils, monocytes, T cells, and B cells. Other chemical cues including metabolic intermediates may also play a role. While it is clear that chemokines lead leukocytes to sites of infection, chemokines appear to have different effects on T cells. For example, neutrophils use the chemokine LTB4 as a signal to move directly toward a site of sterile injury (71). In contrast, the effect of chemokines on T cell movement seems to be less directional than LTB4 effects on neutrophils. In lymph nodes, T cells respond to the chemokines CCL21 and CCL19 by high speed random motion (chemokinesis) rather than directional movement (chemotaxis) (21, 58, 72, 73). Within infected tissue, chemokines appear to increase T cell speed (57, 60) but with only a slight bias toward infection foci (24). Interestingly, we found that the pattern of T cell movement in the lung, at least when infection is not present, does not appear to change when chemokine receptor signaling is inhibited (60).

In social insects, direct agent-agent interaction is an easy and effective way to transmit information. Ants use interaction networks to regulate behavior. Each ant can respond to the rate at which it experiences brief antennal contacts, in which one ant smells the other (74), and rates of brief olfactory interactions influence ant behavior (75). For example, we showed that in an active forager population, the rate of encounter with returning ants determines the probability that an outgoing forager leaves the nest to forage (76, 77). This feedback, based on direct ant-ant interaction, matches current foraging activity to the availability of seeds. Another example is ant-ant interaction leading to regulation of density. It seems that an ant can adjust its movement pattern in response to encountering another ant (78). We found that this change in motion regulates the density of ants in a specific area, enabling ants to spread out if they are too crowded. Rate sensing in ants through direct ant-ant communication provides an additional level of regulation to enhance foraging success. Similarly, direct bee-bee interaction has also been demonstrated to downregulate recruitment to less preferred food locations (79, 80).

It is currently not known whether T cells searching for pathogen infected cells use direct T cell-T cell contact as a mechanism to detect cell density or signal target location. Heterologous cell contacts in the immune response are clearly important, for example, direct contact between T cells and DCs, and T cell-B cell interactions are crucial for an immune response. However, a potential role for homologous cell-cell contact such as T cell-T cell interaction has not been carefully investigated. T cell-T cell interactions have been shown to be important for downregulation of the T cell response through fratricide: Fas-FasL interactions between effector T cells can lead to fratricidal T cell killing, effectively downregulating the T cell response as antigen load decreases (81). Direct T cell-T cell interactions were recently shown to be important in the first phases of T cell activation (82). In the context of T cell response in tissues, little is known about whether T cell—T cell interaction might impact T cell movement.

Thus, although T cells are capable of generating and responding to indirect communication via chemokines and cytokines and direct cell-cell contact with other T cells, it is unclear what the role of direct and indirect communication is in effector T cell search for infected cells (or tumors) in peripheral tissue. Our understanding of search in ants suggests that T cells might use both chemokine-cytokine communication as well as direct cell-cell communication to lead T cells to sites of infection, while also balancing this exploitation of known infection locations with exploration to find new sites of infection.

### Effective Search in Unknown Environments Requires Complex Search Strategies

We illustrate how ant search strategies may vary with the spatial location of resources with a computational model, comparing four foraging strategies in ants (Figure 2): (1) CRW alone (CRW-pink), (2) CRW combined with pheromone recruitment to previously found clusters (pheromone-orange), (3) CRW combined with site fidelity (each individual forager returns to the cluster that it previously found)(site fidelity-blue), and (4) an adaptive strategy known as the Central Place Foraging Algorithm, CPFA, (43) (CPFA-green). CPFA incorporates CRW, site fidelity, pheromone recruitment and the ability to choose among these behaviors based on the density of targets that the searcher senses in the locations immediately adjacent to the searcher.

The CPFA and the foraging model are described in more detail in Hecker and Moses (39). The model represents ants as points that move through space (without collisions and able to detect targets only in the cell in which it is located in and those directly adjacent), and seeds are represented as as points in a grid cell. All ants start at a central nest location, search using the specified strategy for 1 h, and each ant returns each individual seed that it finds directly to the nest (which is at a location known by every ant), carrying one seed at a time. The model uses unitless representations of velocity and length, and the size of the search area was chosen so that complete collection of all seeds is possible in the 1-pile case (Figure 2, column 1).

Figure 2 shows the percentage of the 1,280 seeds that are collected for each spatial distribution. Each search strategy is tested on each of the spatial distributions shown in Figure 1. Figure 2 shows the search performance of simulated ants using different strategies to search for different target distributions. The box plots show the median and interquartile range of 100 replicates for each target distribution, with the seeds placed at random locations drawn from the specified distribution. Where notches in the box plot overlap, the results are statistically indistinguishable (as is the case for pheromone and CPFA in the 1 pile case and randomly dispersed case; all other comparisons are statistically different at the *p* = 0.05 level). As the spatial distribution of targets varies from being concentrated in a single cluster (Figure 1, 1 × 1280) to being more dispersed (Figure 1, 1280 × 1), pheromones become less valuable (compare Figure 2 “pheromone” vs. “CRW” from 1 × 1280 to 1280 × 1). When targets are dispersed at uniform random (1280 × 1), pheromones provide no benefit to foraging at all (and are actually detrimental as they attract ants to locations where a target once was but has been removed). Site fidelity is consistently more effective than random search alone unless resources are completely dispersed, in which case random search is the best strategy.

In the CPFA, searchers decide whether to use random search, site fidelity or pheromone recruitment to a location based on how many targets are there. In highly clustered situations, the CPFA and pheromone are similar in target identification efficiency (1 × 1280), because the CPFA selects a search strategy that relies almost entirely on pheromone search (39). However, the ability of agents to assess and adapt to the environment and choose the appropriate foraging strategy in the CPFA is particularly effective when resources are clustered in many intermediate size piles (16 × 80 or 80 × 16) or in piles with variable sizes such as the power law (compare pheromone and CPFA efficiency).

This search model supports the hypothesis that observed types of directed and random motion in searchers reflect differences in how targets are distributed in different environments. The results in Figure 2 show how different search strategies perform in a fast detection task when searching for static targets. The CPFA has been shown to be effective at collecting up to ~90% of static targets, but ineffective at complete collection (43), particularly when targets are dispersed. Although efficient strategies for complete search or search for mobile or replicating targets may be different (39, 52, 53), our model demonstrates that effective searchers require both a variety of search behaviors and the ability to sense the environment to determine which type of search behavior is best to use in a given time and place.

## Conclusions for T Cell Search

Each ant species is tailored to the particular habitat in which it evolved, but T cells search in wide variety of tissues for a wide variety of targets. T cells demonstrate a variety of search behaviors, including directional movement using chemokine gradients, random motion using CRW, and movement along physical networks. As T cells do not know *a priori* about target distribution and require the capacity to counter unknown future threats, this adaptation and scalability for search in multiple tissues may be particularly important for maintaining effective immunity. Very little is currently known about how effector or memory T cell subsets move in infected tissue. Our observations and models of ants suggest the possibility that effector T cells move directionally toward infected areas in some circumstances (possibly following chemokine gradients, or more speculatively, responding to direct cell-cell contact) and move randomly in others, e.g., to search larger areas when infection has spread broadly or during the memory phase. We hypothesize that different classes of T cells (e.g., central memory cells, tissue resident memory cells and effector cells) have evolved different patterns of movement and responses to external signals and structures, varying with different search goals and target distributions in space and time.

In contrast to T cells moving randomly in tissues, neutrophils appear to move in a highly directed manner toward sites of infection (71). Neutrophils are rapidly recruited to sites of infection, so there is high fidelity between the actual location of infection and the signals, such as chemokines and cytokines, that are produced at sites of infection. Neutrophils moving directionally to foci of infection could be exploiting the close link between timing and spatial distribution early in an infection. The T cell response, on the other hand, develops over many days, with T cells often entering sites of infection 3–5 days post infection. Spatial distribution of the pathogen and related signals may no longer be spatially contained, as earlier in the infection cycle. We suggest that different immune cells (for example, T cells and neutrophils) respond differently to chemokine signals to promote effective immunity at different phases of the immune response with potentially different target distributions.

Understanding the parallels between search strategies in ants and T cells helps illuminate one of the central themes in immunology: how the enormously complex system of trillions of cells, signals, and structures are self-organized into a coherent immune response. As ants and ant search strategies have been studied in detail both experimentally and computationally, we have identified key concepts from ant foraging that suggest new concepts for understanding T cell search to clear infection. Like ants, T cells incorporate many strategies, including directional and random movement, direct agent-agent contact, and use of physical structures. Our proposal is similar to the “No Free Lunch” theorems (83), which posit that there is no single best search or optimization strategy for all computational problems, but that specific solutions can be tailored to specific types of search problems. We posit that there is no one best search strategy that can be used for all search problems in the immune system; instead searchers change how they move and interact with each other and the physical environment in response to specific search problems in specific environments.

Ant foraging strategies have served as inspiration for search heuristics in computer science (70) and as a model of search in a wide variety of complex adaptive systems (39, 84, 85). As we review here, there is increasing evidence that there is no single effective ant search strategy, but rather a repertoire of search behaviors that includes varied ways of moving, communicating, and using environmental structures to form an effective response to environmental conditions. Understanding the multiple components of effective ant search, and how they are combined into different strategies to respond to varied and dynamic environments can translate to new approaches for understanding the even more complex search processes of the immune system.

## Author Contributions

MM, JC, DG, and SF conceived of the ideas for the article. MM, JC, DG, and SF contributed to the writing and editing of the article. MM generated the figures.

### Conflict of Interest Statement

The authors declare that the research was conducted in the absence of any commercial or financial relationships that could be construed as a potential conflict of interest.
